# Geostatistical Analysis of the Variability in *Sthenoteuthis oualaniensis* Fishing Grounds in the Northwestern Indian Ocean High Seas

**DOI:** 10.3390/ani16030393

**Published:** 2026-01-27

**Authors:** Ruizhi Zhou, Hanfeng Zheng, Yongchuang Shi, Lingzhi Li, Wei Fan, Ziniu Li, Guoqing Zhao, Fenghua Tang

**Affiliations:** 1 East China Sea Fisheries Research Institute, Chinese Academy of Fishery Sciences, Shanghai 200090, China; zrz17638310406@163.com (R.Z.); zhenghf@ecsf.ac.cn (H.Z.); syc13052326091@163.com (Y.S.); lilz@ecsf.ac.cn (L.L.); fanwee@126.com (W.F.); lizn@ecsf.ac.cn (Z.L.); 2 College of Marine Sciences and Ecology, Shanghai Ocean University, Shanghai 201306, China

**Keywords:** *Sthenoteuthis oualaniensis*, northwest Indian Ocean high seas, spatial autocorrelation, standard deviation ellipse, hotspot analysis, GAM

## Abstract

*Sthenoteuthis oualaniensis* is a major commercial species in the high-seas fisheries of the northwestern Indian Ocean. However, its spatiotemporal distribution exhibits strong uncertainty under climate and environmental variability, complicating the understanding of fishing ground dynamics. To investigate the spatiotemporal distribution of *S. oualaniensis* under climate change, this study analyzed commercial fishing data from 2016 to 2024. The results indicate that the core distribution area of the species is consistently concentrated within 14–19° N and 61–65° E. From 2016 to 2024, the fishing ground expanded annually and shifted overall toward the east and north, with its centroid showing a persistent northeastward trajectory. Global spatial autocorrelation analysis revealed positive and significant Moran’s I values for all years, demonstrating a strong spatial clustering pattern. Hotspot analysis shows that high-abundance areas were primarily located north of 14° N, with an overall northeastward migration trend. Hotspots expanded continuously from 2016 to 2023, but sharply contracted in 2024, shifting further northeast and becoming restricted to 63–68° E and 19–21° N. The GAM results indicate that CPUE in the region is jointly influenced by spatiotemporal drivers and multiple environmental factors. These findings confirm that the spatiotemporal distribution and population dynamics of *S. oualaniensis* are shaped by the combined effects of environmental variability and temporal–spatial factors, with environmental influences playing a particularly crucial role.

## Simple Summary

*Sthenoteuthis oualaniensis* is a key commercial species in the high-seas fisheries of the northwestern Indian Ocean. Its spatiotemporal distribution is highly uncertain due to the combined influence of climate variability and environmental changes, making fishing ground prediction challenging. In this study, spatial autocorrelation analysis and the standard deviation ellipse model were employed to elucidate the spatiotemporal dynamics of *S. oualaniensis* fishing grounds, and a GAM model was used to quantify the relationships between its abundance and environmental factors. The results show that the fishing ground centroid has shifted progressively eastward and northward, the resource distribution exhibits clear spatial clustering, and the hotspot areas have expanded toward the northeast. Spatiotemporal factors exert significant impacts on the distribution of *S. oualaniensis*. Overall, this study highlights the integrative regulatory effects of multi-scale oceanographic variability on the species’ spatial distribution and provides scientific support for dynamic management and sustainable utilization of high-seas fishing grounds in the northwestern Indian Ocean.

## 1. Introduction

The Northwest Indian Ocean (NWIO) is a typical monsoon-driven marine region, where oceanographic conditions are strongly regulated by seasonal monsoon systems [1,2]. In the Arabian Sea and adjacent high-seas areas, the southwest monsoon during summer drives the development of the Somali upwelling and mesoscale eddies—such as the Great Whirl—which transport nutrient-rich deep waters into the euphotic zone, substantially enhancing primary productivity [3,4,5]. This process forms a distinct “physical–biological coupling” mechanism, endowing the region with exceptionally high biological carrying capacity and making it an important foraging ground for various oceanic migratory fishes and cephalopods. Against the backdrop of global declines in coastal fishery resources, the NWIO high seas have become a hotspot for international distant-water fisheries, particularly for cephalopod resources that are not subject to strict quota regulations and thus hold considerable development potential.

*Sthenoteuthis oualaniensis*—commonly known as the purpleback flying squid—is one of the dominant cephalopod species in the NWIO [6]. As a typical warm-water oceanic species, *S. oualaniensis* exhibits classic “r-strategy” life-history traits, including a short lifespan, rapid growth, high fecundity, and fast generational turnover [7]. Within the marine food-web structure, this species acts as both a key prey item for large migratory predators such as tunas and an active secondary predator itself, functioning as an important ecological nexus [8]. However, due to its short-lived and “ecological opportunist” nature, the abundance and spatial distribution of *S. oualaniensis* are highly sensitive to environmental variability, resulting in pronounced interannual fluctuations in fishery yields [9].

Existing studies have demonstrated that environmental factors such as sea surface temperature (SST), sea surface chlorophyll a concentration (Chl-a), and sea surface height (SSH) are key determinants shaping the formation of *S. oualaniensis* fishing grounds [10,11]. However, under global warming, the continuous increase in Indian Ocean SST, together with the frequent occurrence of large-scale climate modes such as the El Niño–Southern Oscillation (ENSO) and the Indian Ocean Dipole (IOD), has profoundly altered the region’s thermodynamic structure and the distribution of feeding grounds [12]. Clarifying the complex linkages between these environmental variables and fluctuations in fishery yield is essential for the scientific development and adaptive management of this resource.

Accurately characterizing spatial distribution patterns is central to fishery management. Traditional linear regression models are limited in capturing the nonlinear threshold effects of environmental variables on biological resources [13]. Although generalized additive models (GAMs) can effectively account for complex nonlinear relationships, they typically assume sample independence and often neglect the intrinsic spatial autocorrelation inherent in fishery data [14,15]. As a species that exhibits pronounced aggregation behavior, *S. oualaniensis* tends to form highly clustered high-yield areas. Ignoring such spatial autocorrelation may lead to biased parameter estimates and reduced statistical power. Therefore, integrating spatial autocorrelation analysis with GAM is crucial for disentangling fishing-ground patterns from the dual perspectives of environmental forcing and spatial structure.

Given this context, the present study combines commercial fishing production data (2016–2024) with multi-source satellite-derived environmental datasets to achieve the following objectives: (1) Using spatial autocorrelation analysis to investigate the spatial aggregation patterns of *S. oualaniensis* resource abundance and the distribution of hot and cold spots; (2) to develop a GAM framework incorporating spatial information to quantitatively assess the nonlinear regulatory effects of key environmental factors on *S. oualaniensis* distribution; and (3) to reveal the spatiotemporal dynamics of *S. oualaniensis* fishing grounds in the NWIO under ongoing climate change. The findings of this study will provide scientific support for precise fishing-ground forecasting and sustainable management of *S. oualaniensis* resources in the NWIO high seas.

## 2. Materials and Methods

### 2.1. Data Sources, Pre-Processing, and Fishing Ground Area

**Figure 1:**
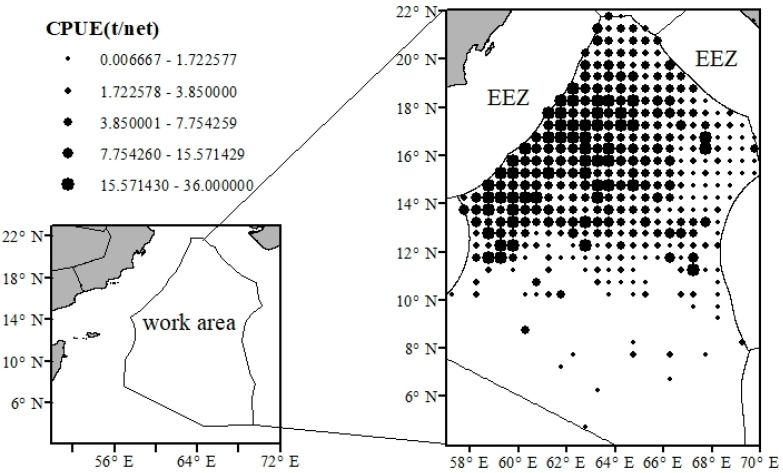
Schematic map of the study area for *S. oualaniensis* in the northwestern Indian Ocean during 2016–2024. Note: EEZ denotes the Exclusive Economic Zone; the study area extends from 4° N to 22° N and 57° E to 70° E. Black circles indicate CPUE values aggregated for each latitude–longitude grid cell. The size of the circles is proportional to CPUE, with larger circles representing higher CPUE levels at the corresponding locations. The inset map shows the location of the study area.

The fishery data used in this study were derived from the statistical records of China’s distant-water fisheries, covering commercial fishing operations for *S. oualaniensis* in the high seas of the Northwest Indian Ocean (4–22° N, 57–70° E) from 2016 to 2024 (Figure 1). The original fishery statistics include information on fishing time (year and month), vessel positions (latitude and longitude), number of fishing operations (sets), and catch. Among these, the number of fishing operations refers to the total count of fishing activities conducted within a given temporal scale (annual or monthly) and a defined spatial domain (fishing grids delineated by latitude and longitude). Catch represents the total production of *Sthenoteuthis oualaniensis* harvested within the corresponding spatiotemporal grid. To achieve spatial standardization, the study area was divided into 0.5° × 0.5° grid cells. The catch per unit effort (CPUE) within each grid was calculated to characterize the spatial distribution patterns of *S. oualaniensis* abundance, and the spatiotemporal distribution of fishing grounds was visualized using ArcGIS 10.8.2 software. CPUE is a primary index in fishery stock assessments, generally assumed to be proportional to stock abundance [16]. CPUE was calculated as CPUE = C/E, where C represents the total catch of *Sthenoteuthis oualaniensis* (tons) within a unit fishing grid defined by latitude and longitude, and E denotes the total number of fishing operations (nets) conducted within the corresponding spatial grid [17]. In addition, to account for the contribution of each fishing operation to fishing-ground dynamics, yield-based point features were used in the spatial autocorrelation analysis, standard deviational ellipse analysis, and catch gravity-center analysis [18].

Previous studies have indicated that Sea Surface Temperature (SST), Sea Surface Salinity (SSS), Chlorophyll-a concentration (Chl-a), Sea Surface Height (SSH), and sea surface geostrophic currents (comprising the northward component-VO, and eastward component-UO) significantly influence the distribution of *S. oualaniensis* [19]. To investigate the environmental drivers of fishing ground distribution, multi-source marine environmental remote sensing data were selected for this study. These environmental datasets were obtained from the Copernicus Marine Service (https://resources.marine.copernicus.eu/products, accessed on 30 October 2025). The data were processed to match the spatiotemporal resolution of the fishery data, specifically at a monthly temporal scale. All geographical distribution maps and spatial autocorrelation plots were generated using ArcGIS software.

### 2.2. Global Spatial Autocorrelation

Global spatial autocorrelation describes the spatial characteristics of attribute values across an entire region. It measures the degree of association among spatial objects to determine whether significant spatial distribution patterns exist. Common statistical metrics used for this analysis include the Global Moran’s, Global Geary’s *C*, and Global Getis-Ord *G* [20]. In this study, the Global Moran’s *I* was adopted to quantify the overall spatial autocorrelation of *S. oualaniensis* catches, and the standardized Z-score was used to test the significance of the index. The calculation formula is as follows [21]:

(1) $$I=\frac{n\sum_{i=1}^{n}\sum_{j=1}^{m}w_{ij}\left(x_{i}-\bar{x}\right)\left(x_{j}-\bar{x}\right)}{\sum_{i=1}^{n}\sum_{j=1}^{m}w_{ij}{\left(x_{i}-\bar{x}\right)}^{2}}(i\neq j)$$

(2) $$Z_{\mathrm{score}}=\frac{I-E(I)}{\sqrt{\mathrm{VAR}(I)}}$$

In the formula, *I* represents the Global Moran’s *I* index; n denotes the total number of spatial units analyzed; $x_{i}$ and $x_{j}$ represent the resource density at locations *i* and *j*, respectively; and *w_ij_* is the spatial weight coefficient characterizing the spatial relationship between units *i* and *j* (*w_ij_ =* 1 if adjacent, otherwise *w_ij_ =* 0). $\bar{x}\;$denotes the mean value of all units, while *E(I)* and *VAR(I)* represent the expected value and variance of Moran’s *I*, respectively. The Global Moran’s *I* ranges from −1 to 1 and serves to test the null hypothesis of spatial randomness within the study area [22]. Values of *I > 0* indicate positive spatial autocorrelation, with higher values implying stronger clustering. Conversely, *I* < 0 indicates negative spatial autocorrelation, suggesting spatial dispersion or heterogeneity. A value of zero or near-zero indicates no spatial autocorrelation (i.e., a random distribution). Significance testing for Moran’s *I* relies on the *Z*-score and *p*-value. The *Z*-score represents the number of standard deviations by which the observed *I* deviates from the expected value; a highly positive *Z*-score indicates significant clustering, while a negative *Z* -score suggests dispersion. The *p*-value assesses the probability that the observed spatial pattern arose by random chance; a low *p*-value (typically *p* < 0.05) indicates statistical significance [23]. In this study, all computations were performed using GeoDa 1.14.0, employing 99,999 random permutations to ensure the robustness of the significance test.

### 2.3. Local Spatial Autocorrelation

While global spatial autocorrelation quantifies the overall degree of clustering across the entire study area, it fails to effectively reveal local spatial associations between specific spatial units and their neighbors. To address this limitation, this study introduced a local spatial autocorrelation indicator—the Getis-Ord Gi* statistic (hotspot analysis)—to identify local clustering characteristics. This method detects statistically significant clusters of high values (hotspots) and low values (cold spots). Consequently, it was applied to pinpoint specific areas where high (or low) *S. oualaniensis* catches are aggregated. The calculation formula for Getis-Ord Gi* is as follows [24]:

(3) $$Gi*=\frac{\sum_{j=1}^{n}w_{i,j}x_{j}-\bar{X}\sum_{j=1}^{n}w_{i,j}}{s\times \sqrt{n\left(\sum_{j=1}^{n}w_{i,j}^{2}-{\left(\sum_{j=1}^{n}w_{i,j}\right)}^{2}\right)/\left(n-1\right)}}$$

In the formula, $x_{j}$ represents the attribute value of feature j; $w_{i,j}$ denotes the spatial weight between features *i* and j (equal to 1 if adjacent and 0 otherwise); n is the total number of sample points; and $\bar{x}$ and *S* represent the mean and standard deviation, respectively. The Gi* statistic yields a Gi* Z-score and a *p*-value, whose interpretations are similar to those of the Z-score and *p*-value derived from the global spatial autocorrelation analysis. When the Gi* Z-score exceeds ±2.58 standard deviations with *p* < 0.01, the feature is identified as a statistically significant hotspot (or cold spot). When the Gi* Z-score ranges from 1.96 to 2.58 (or −2.58 to −1.96) standard deviations with *p* > 0.05, the resource may exhibit a potential hotspot (or cold spot) pattern, although a random distribution cannot be ruled out. A Gi* Z-score between 1.65 and 1.96 (or −1.96 to −1.65) with *p* > 0.01 indicates that the resource is highly likely to follow a random distribution. When the Gi* Z-score falls within ±1.65 standard deviations, the resource is considered to be randomly distributed within the study area [18,25].

### 2.4. Migration Trajectory of the Center of Gravity and Standard Deviation Ellipse Analysis

The migration trajectory model of the center of gravity can represent the spatiotemporal movement patterns of geographic features based on their weighted centers [26]. In this study, the catch of *S. oualaniensis* was used as the weighting variable to calculate the temporal and spatial variation in the fishing ground’s center of gravity. The standard deviation ellipse is a spatial statistical method used to quantitatively describe the overall characteristics and spatiotemporal evolution of the spatial distribution of geographic features. It uses the mean center, major axis, minor axis, and azimuth as key parameters, and its results can effectively capture the centrality, dispersion, directional trend, and shape of the spatial distribution [27]. In this study, catch was used as the weighting field to reveal the directional variation and spatial distribution patterns of *S. oualaniensis* fishing grounds. The relevant parameter calculation formulas are as follows:

(4) $${\bar{X}}_{w}\;=\frac{\sum_{i=1}^{n}(C_{i}\times X_{i})}{\sum_{i=1}^{n}C_{i}};\;{\bar{Y}}_{w}=\frac{\sum_{i=1}^{n}(C_{i}\times Y_{i})}{\sum_{i=1}^{n}C_{i}}$$

(5) $$\tan\theta =\frac{\left(\sum_{i=1}^{n}{w_{i}}^{2}{{\tilde{x}}_{i}}^{2}-\sum_{i=1}^{n}{w_{i}}^{2}{{\tilde{y}}_{i}}^{2}\right)+\sqrt{\sum_{i=1}^{n}{w_{i}}^{2}{{\tilde{x}}_{i}}^{2}-\sum_{i=1}^{n}{w_{i}}^{2}{{\tilde{y}}_{i}}^{2}-4\sum_{i=1}^{n}{w_{i}}^{2}{{\tilde{x}}_{i}}^{2}{{\tilde{y}}_{i}}^{2}}}{2\sum_{i=1}^{n}{w_{i}}^{2}{\tilde{x}}_{i}{\tilde{y}}_{i}}$$

(6) $$\delta_{x}=\sqrt{\frac{\sum_{i=1}^{n}{\left(w_{i}{\tilde{x}}_{i}\cos\theta -w_{i}y_{i}\sin\theta \right)}^{2}}{\sum_{i=1}^{n}{w_{i}}^{2}}}$$

(7) $$\delta_{y}=\sqrt{\frac{\sum_{i=1}^{n}{\left(w_{i}{\tilde{x}}_{i}\sin\theta -w_{i}y_{i}\cos\theta \right)}^{2}}{\sum_{i=1}^{n}{w_{i}}^{2}}}$$

In the formulas, ${\bar{X}}_{w}$ and$\;{\bar{Y}}_{w}$ represent the coordinates of the center of gravity of the *S. oualaniensis* fishing grounds; $C_{i}$ is the catch of the *i*-th haul; $X_{i}$ and $Y_{i}$ denote the latitude and longitude of the *i*-th haul, respectively; and *n* is the total number of hauls during the study period. *θ* is the azimuth of the ellipse; $w_{i}$ is the weight of each spatial unit; $x_{i\;}$and $y_{i}$ represent the centroid coordinates of each spatial unit; ${\tilde{x}}_{i}$ and ${\tilde{y}}_{i}$ denote the deviations of each spatial unit’s centroid from the ellipse’s mean center; and $\delta_{x}$ and $\delta_{y}$ represent the standard deviations along the *x*-axis and *y*-axis, respectively. In the standard deviation ellipse analysis, a one-standard-deviation ellipse was applied.

### 2.5. GAM Analysis

Given that the effects of marine environmental variables on fishery resources often exhibit nonlinear characteristics, GAM provides a flexible framework for describing nonlinear ecological relationships. They allow a variety of error distributions and can incorporate spatial and temporal smoothers, offering strong interpretability and model stability [28,29]. Compared with GLMs and machine learning approaches, GAM maintains model transparency while accurately capturing complex marine ecological processes, making it one of the most suitable methods for analyzing fishery resource distribution and environmental driving mechanisms [30,31]. In this study, GAM was applied to examine the spatiotemporal distribution of *S. oualaniensis* in the northwestern Indian Ocean and its relationships with key environmental factors. In the model, the CPUE of *S. oualaniensis* was used as the response variable, while Year, Longitude, Latitude, Chl-a, SST, SSS, SSH, UO, and VO were included as explanatory variables describing its spatiotemporal distribution. To avoid the undefined nature of logarithmic transformation at zero values and to ensure numerical stability, the response variable was subjected to a log-shift transformation, expressed as log(CPUE + c). Log-transformation reduces the influence of extreme values and promotes approximate normality of residuals, thereby facilitating the application of smooth terms within the GAM framework to characterize the nonlinear effects of environmental factors [29]. Prior to transformation, a small constant (c = 0.1) was added to accommodate zero CPUE values and to maintain numerical stability, consistent with standard practices in fisheries CPUE standardization. The GAM is expressed as follows:

log(CPUE + 0.1)~s(Year) + s(Longitude) + s(Latitude) + s(Chl-a) + s(SST) + s(SSS) + s(SSH) + s(UO) + s(VO)+ ε(8)

In the model, CPUE was treated as the response variable. The term s(Year) represents the temporal explanatory variable “year,” while s(Longitude) and s(Latitude) correspond to the spatial explanatory variables “longitude” and “latitude,” respectively. The smooth terms s(Chl-a), s(SST), s(SSS), s(SSH), s(UO), and s(VO) represent the environmental explanatory variables, including chlorophyll-a concentration, sea surface temperature, sea surface salinity, sea surface height, and the northward and eastward components of geostrophic surface current velocity. The term ε denotes the random error. Variance Inflation Factors (VIFs) were used to assess the independence of the explanatory variables, and the results are presented in Table 1. All predictor variables exhibited acceptable levels of collinearity (VIF < 3), which is consistent with the established diagnostic threshold for regression models [32]. This conservative criterion minimizes the risk of Type I errors while maintaining model parsimony, a practice supported by Monte Carlo simulations in ecological studies [33]. According to the VIF assessment (Table 1), all VIF values were below 3, indicating no severe multicollinearity among the explanatory variables [34].

The model was developed under a stepwise regression framework, in which predictor variables were selected by optimizing the Akaike Information Criterion (AIC) [35] and the proportion of deviance explained [36]. Improvements in model performance were reflected by decreases in AIC values and increases in the explained deviance [32]. The model was implemented using the mgcv package in R 4.4.3.

## 3. Results and Analysis

### 3.1. Changes in Catch

**Figure 2:**
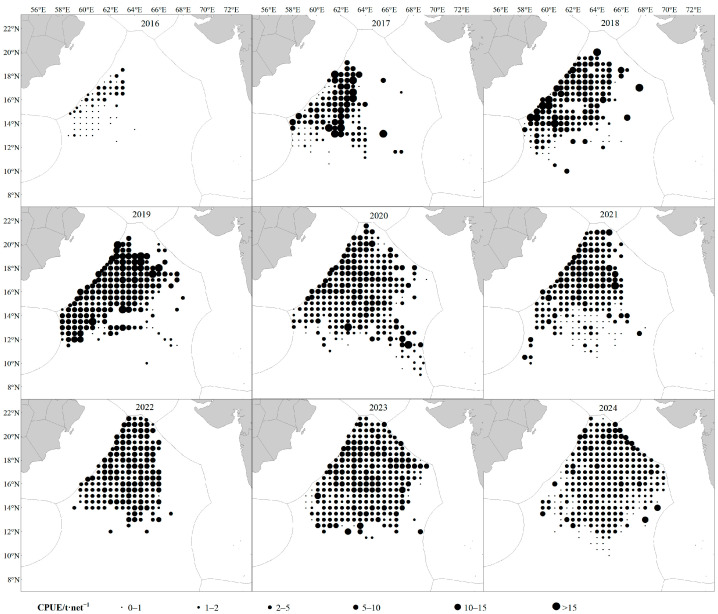
Catches distribution of *S. oualaniensis* in the high seas of the Northwest Indian Ocean from 2016 to 2024.

**Figure 3:**
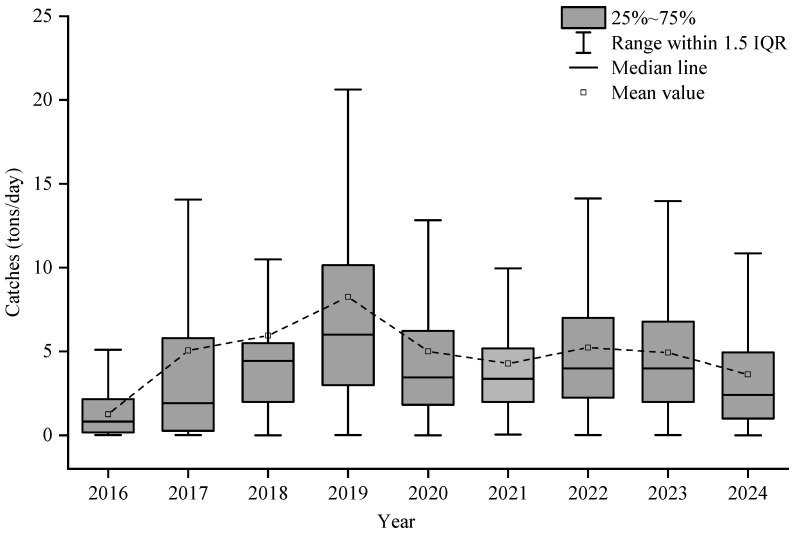
Changes in single-day catches of *S. oualaniensis* in the high seas of the Northwest Indian Ocean from 2016 to 2024.

**Figure 4:**
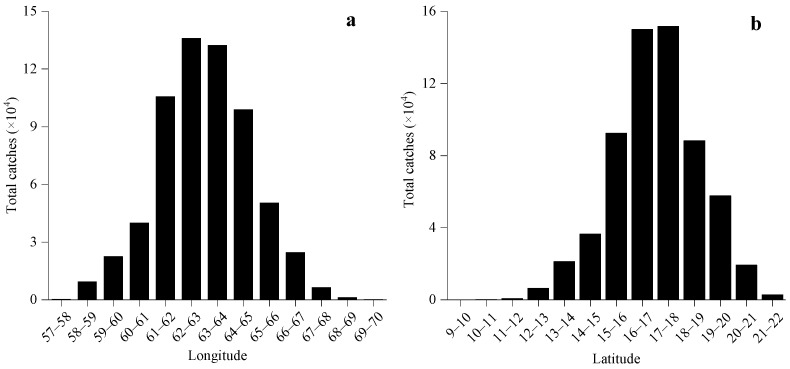
Variation in the *S. oualaniensis* in the high seas of the Northwest Indian Ocean with longitude (**a**) and latitude (**b**) from 2016 to 2024.

From 2016 to 2024, the *S. oualaniensis* fishing grounds in the northwestern Indian Ocean high seas exhibited a gradual expansion, with the distribution area increasing year by year and extending progressively toward the east and north. The spatiotemporal distribution of CPUE showed marked variability. In 2016, CPUE values were generally low, all below 5 t·net^−1^. From 2017 to 2019, the proportion of fishing units with CPUE greater than 5 t·net^−1^ increased annually, reaching a maximum of 72.92% in 2019. Although the fishing grounds continued to expand thereafter, CPUE displayed a decreasing trend (Figure 2). Daily catch exhibited pronounced interannual fluctuations, showing an overall pattern of increasing first, then decreasing, and finally stabilizing. The maximum value occurred in 2019, after which variability declined (Figure 3). Spatially, high-yield areas were mainly concentrated within the longitude range of 61–65° E, accounting for 75.43%, and the latitude range of 14–19° N, accounting for 76.97% (Figure 4).

### 3.2. Environmental Factor Contributions

**Figure 5:**
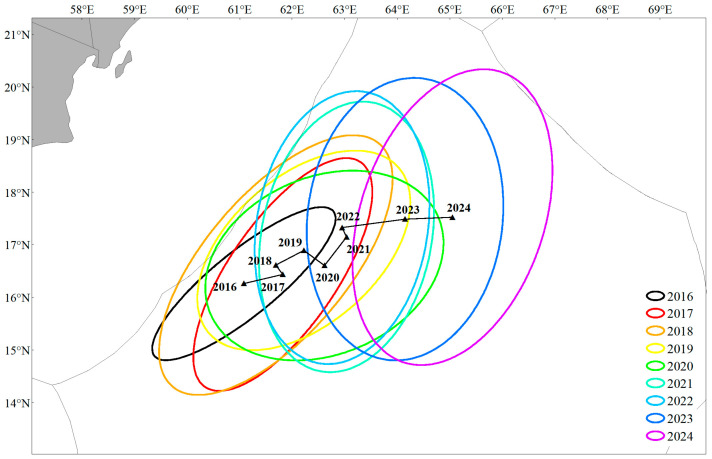
Changes in the center of gravity and SDE of the fishing grounds from 2016 to 2024.

**Figure 6:**
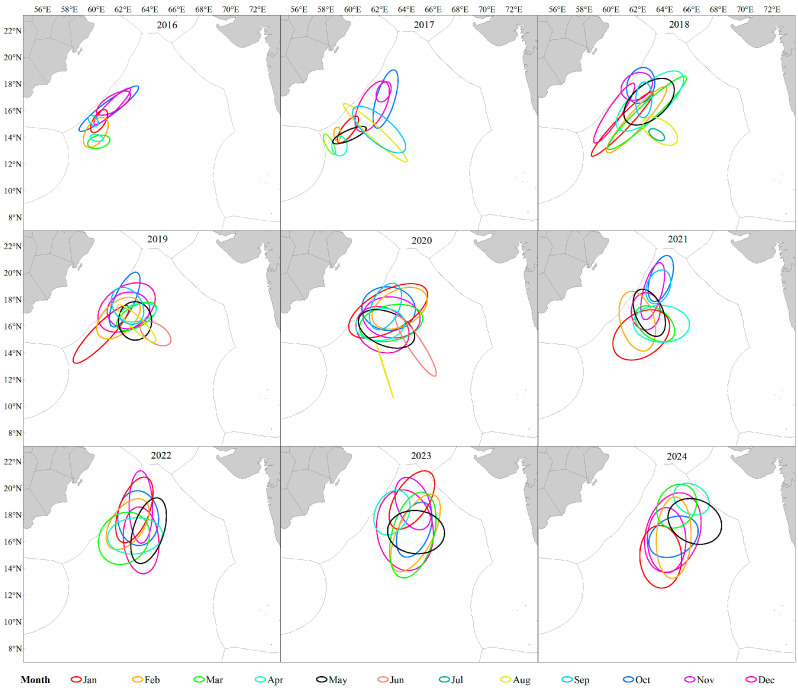
Seasonal variation in the gravity center of the fishing grounds in the northwestern Indian Ocean during 2016–2024.

From 2016 to 2024, the centroid of the *S. oualaniensis* fishing grounds in the northwestern Indian Ocean exhibited a distinct northeastward migration trend. The Standard Deviation Ellipse (SDE) analysis indicated a significant expansion of the fishing grounds, with the dominant expansion axis gradually shifting from an initial southwest–northeast orientation to a nearly north–south pattern (Figure 5). At the monthly scale, both the extent and direction of centroid expansion showed considerable interannual variability, without a clear or consistent seasonal migration pattern (Figure 6). This suggests that the *S. oualaniensis* population in this region may be resident or capable of only short-distance movements. Nevertheless, the monthly centroid trajectories displayed a general southwest–northeast seasonal shift. From January to May, the fishing-ground centroid was mainly concentrated in the southwestern area; from June to October, it tended to migrate northeastward; and from November to December, the northward movement weakened, with noticeable southward retreat observed in certain years.

### 3.3. Global Spatial Autocorrelation and Descriptive Statistics

To obtain an overview of the *S. oualaniensis* resource status, descriptive statistics and global spatial autocorrelation analyses were conducted (Table 2). From 2016 to 2024, all skewness (Sk) values were greater than zero, indicating positively skewed distributions in which low-catch areas dominated. Except for 2016 (Ku = 0.52), the kurtosis (Ku) values in all other years were significantly greater than 3, showing a pronounced leptokurtic pattern with sharp peaks and heavy tails. This pattern was particularly extreme in 2020 (Ku = 110.04), suggesting the presence of exceptionally high-yield aggregation hotspots during that year. The coefficient of variation (CV) remained consistently high (0.82–1.48), indicating strong spatial heterogeneity in the distribution of *S. oualaniensis* resources. Global Moran’s I results showed that *S. oualaniensis* exhibited varying degrees of positive global spatial autocorrelation across all years in the northwestern Indian Ocean high seas. In addition, *Z*-scores for all years exceeded 2.58 and *p*-values were <0.0001, demonstrating a statistically significant clustered distribution pattern from 2016 to 2024.

### 3.4. Hotspot and Coldspot Distribution

**Figure 7:**
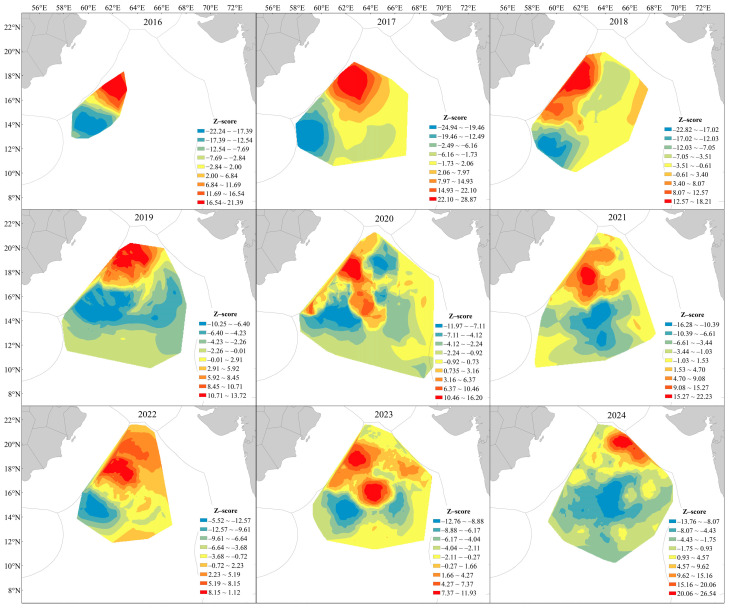
Hotspots of annual catch of *S. oualaniensis* in the Northwest Indian Ocean.

From 2016 to 2024, both hotspot and coldspot areas of *S. oualaniensis* exhibited clear spatial clustering patterns with pronounced spatiotemporal variability (Figure 7). Between 2016 and 2018, hotspots were highly concentrated in the northwestern region (approximately 15–20° N, 60–66° E), while coldspots were consistently distributed along the southern and southwestern margins. In 2019, hotspots remained in the northern area, but coldspots began to extend toward the central region. In 2020, hotspots and coldspots overlapped extensively. In 2021, the hotspots became re-concentrated, shifting toward the western portion of the study area. From 2022 to 2024, hotspots re-emerged and migrated northward, with a notable increase in coldspots in 2024. Overall, hotspot areas were consistently located north of 14° N, with a general tendency to shift northeastward. Hotspot regions expanded from 2016 to 2023 but contracted sharply in 2024 while continuing to move further northeastward, occupying the zone between 63–68° E and 19–21° N.

### 3.5. Analysis of GAM Results

#### 3.5.1. GAM Diagnostics

As summarized in Table 3, the optimal GAM—log(CPUE + 0.1)~s(Year) + s(Longitude) + s(Latitude) + s(Chl-a) + s(SST) + s(SSS) + s(SSH) + s(UO) + s(VO)—exhibited strong model performance, with an AIC of 3962.039, 54.4% deviance explained, and an R^2^ value of 0.537. The statistical significance of each variable in the final optimized GAM is presented in Table 4. According to the F-test results, the relative contributions of the spatiotemporal and environmental predictors to the model, from highest to lowest, were: Year, Latitude, SSS, SST, Longitude, UO, Chl-a, VO, and SSH. All predictor variables showed significant statistical associations with CPUE (*p* < 0.0001). Together, these results indicate that the iteratively optimized GAM effectively avoids overfitting and reliably captures the underlying structure of the data.

#### 3.5.2. Relationship Between CPUE and Environmental/Spatiotemporal Factors

**Figure 8:**
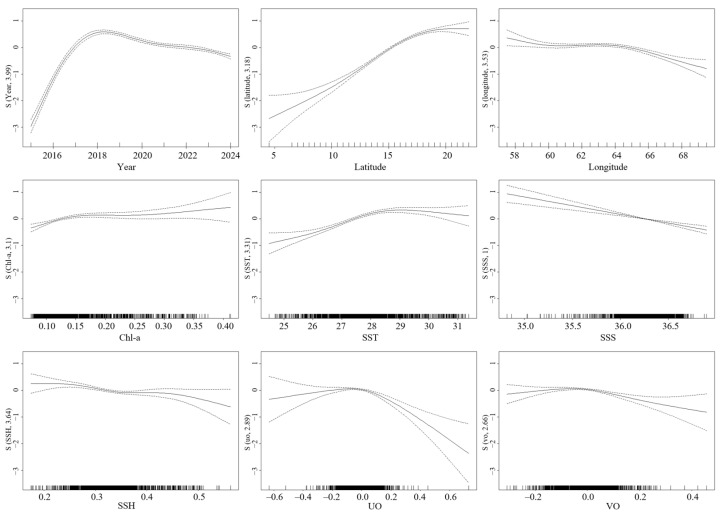
The impact of each explanatory variable on the CPUE of *S. oualaniensis* in the Northwest Indian Ocean. Note: The solid line in the middle represents the fitted result of the model, reflecting the smooth relationship between variables. The upper dashed line represents the upper confidence interval of the model, indicating the upper limit of the model’s estimate. The lower dashed line represents the lower confidence interval of the model, indicating the lower limit of the model’s estimate.

The stock abundance of *S. oualaniensis* shows complex relationships with multiple environmental and spatiotemporal factors in the fishing grounds (Figure 8). Using CPUE as the response variable for stock abundance, the figure illustrates the nonlinear partial effects of nine explanatory variables on CPUE. Regarding spatiotemporal factors, the year effect indicated that stock abundance initially increased and then slowly decreased from 2016 to 2024, peaking in 2018. The response curves for spatial variables revealed that high-density areas were primarily concentrated in the Northwest Indian Ocean (15–22° N, 58–65° E). Environmental factors exhibited varying effects. Chl-a concentration showed a positive relationship with CPUE, with a pronounced increase in CPUE when Chl-a exceeded 0.15–0.2 mg/m^3^. CPUE remained relatively stable when SST was within the threshold range of 28–30 °C but declined outside this range. CPUE was negatively correlated with SSS and SSH. CPUE reached its highest values when both UO and VO were close to 0 m/s, while stronger currents (i.e., absolute values > 0) had negative effects.

## 4. Discussion

### 4.1. Spatiotemporal Distribution Characteristics of S. oualaniensis Resources

In the northwestern Indian Ocean, the strong upwelling and cold-eddy activity driven by the southwest monsoon transport nutrient-rich deep water into the euphotic zone, thereby stimulating intense phytoplankton primary production [3,5]. As the core region of the tropical Indian Ocean, the Arabian Sea’s semi-enclosed geographic configuration, together with the surrounding hot and arid climate, forms a unique marine environment characterized by elevated sea surface temperatures [37]. According to the trophic cascade effect in marine ecosystems, the outbreak of primary productivity is rapidly transmitted through the food web, attracting large numbers of small epipelagic fishes and crustaceans that feed on zooplankton. This trophic enhancement ultimately provides abundant prey resources for *S. oualaniensis*, a high–trophic level species [38].

Based on CPUE and environmental data from the northwestern Indian Ocean high seas during 2016–2024, this study reveals pronounced spatiotemporal heterogeneity in the distribution of *S. oualaniensis* resources. Temporally, CPUE was markedly low in 2016 (less than 5 t/net), but exhibited a continuous increasing trend from 2017 to 2024 (Figure 2). This pattern may be associated with the strong El Niño event in 2016, which caused substantial oceanographic anomalies [39]. During ENSO events, the associated atmospheric circulation anomalies alter evaporation and cloud cover, subsequently modifying the net heat flux entering remote ocean regions. These heat flux anomalies induce significant sea-surface temperature deviations, leading to warmer-than-normal conditions across the Indian Ocean [40]. Spatially, the high-yield fishing grounds were consistently concentrated between 61–65° E and 14–19° N, with longitude and latitude contributing 75.43% and 76.97% of the total CPUE, respectively (Figure 4). This core region aligns closely with the extension of the Somali upwelling system and the high-productivity frontal zone along the periphery of the Great Whirl in the Arabian Sea. Such correspondence highlights the critical role of sustained nutrient input in maintaining the dominance of this fishing ground [41].

Furthermore, the GAM model elucidated the nonlinear regulatory mechanisms of environmental variables on the spatial distribution of *S. oualaniensis*. The results show that CPUE is strongly positively correlated with Chl-a, but negatively correlated with SSH and SSS. This combination of high Chl-a and low SSH is a typical signature of cyclonic eddies or upwelling regions, indicating that *S. oualaniensis* tends to inhabit highly productive areas characterized by intensified eddy activity or strong upward nutrient transport [42]. Previous studies have demonstrated that cyclonic eddies enhance surface productivity by uplifting deep nutrient-rich waters into the euphotic zone, thereby increasing Chl-a concentrations [43]. In addition, eddies can modify the spatial patterns of temperature and nutrient distributions, indirectly influencing the abundance and habitat range of cephalopod populations [44]. The positive relationship between SST and CPUE further supports the physiological characteristics of *S. oualaniensis* as a warm-water oceanic species [38]. In contrast, the negative correlation between SSS and CPUE suggests an avoidance of high-salinity water masses and a preference for mixed water influenced by equatorial low-salinity systems. Such specific temperature–salinity preferences may constrain its expansion toward higher latitudes or deeper offshore waters [45]. This study also demonstrates that *S. oualaniensis* in the northwestern Indian Ocean exhibits a distinct preference for particular flow field conditions. Both zonal (UO) and meridional (VO) geostrophic current components associated with peak CPUE values were concentrated near 0 m/s. This preference for low-flow environments indicates that the species tends to aggregate in regions with minimal current shear—typically frontal zones at eddy boundaries or weak-current regions [3,5]. Such environments help reduce locomotion energy expenditure while simultaneously enhancing feeding efficiency through the accumulation of prey organisms driven by hydrodynamic convergence [46].

### 4.2. Analysis of Fishing Ground Variability

In fisheries resource research, the fishing ground gravity center is not merely a geographic coordinate but a key indicator reflecting how resources respond to environmental variability [47]. In this study, the gravity center and hotspot areas of *S. oualaniensis* in the northwestern Indian Ocean exhibited a clear southwest–northeast migration trend during 2016–2024, shifting cumulatively by 3.97° E and 1.26° N (Figure 6), accompanied by a progressive expansion of the fishing operation range. Such long-term spatial shifts are likely driven by the combined effects of sustained sea surface warming in the Indian Ocean and interdecadal variations in monsoon patterns under global climate change [48,49]. Marine species commonly track suitable temperature–salinity boundaries through migration to maintain optimal conditions for survival and reproduction [50]. Existing studies have shown that as oceans warm, species tend to shift their suitable habitat boundaries toward higher latitudes in search of favorable thermal environments [51], which is highly consistent with the northward displacement observed in this study. Moreover, the eastward shift of the gravity center may be associated with variations in the strength of the Arabian Sea monsoon circulation. Changes in monsoon intensity can directly alter the position of the Somali upwelling and adjacent upwelling systems, resulting in eastward displacement and consequently driving spatial reconfiguration of prey fields and *S. oualaniensis* distributions [52]. Previous research has suggested that phase changes in large-scale climate modes can modify the spatial structure of upwelling systems, leading to zonal displacement of prey-rich habitats. The eastward expansion trajectory of the fishing grounds observed in this study further supports the concept that *S. oualaniensis* actively tracks the spatial reorganization of its prey fields.

This study reveals that, on an interannual scale, the gravity center of *S. oualaniensis* fishing grounds exhibited an overall northeastward migration trend during 2016–2024 (Figure 5). However, at the monthly scale, the pattern differed: although the spatiotemporal evolution of the gravity center showed certain interannual fluctuations (e.g., a more dispersed pattern in 2022), it generally displayed a “southwest–northeast” oscillatory movement consistent with the direction of the monsoon (Figure 6). This spatial displacement does not indicate long-distance, cross-basin migration; rather, it represents short-distance habitat tracking in response to seasonal environmental variations, reflecting the seasonal dynamics of the Somali upwelling and Arabian Sea eddy activity driven by the southwest monsoon [53,54,55]. The alternating intensification and weakening of monsoon and eddy activities result in a relatively stable, suitable habitat range within the core fishing grounds, with only minor local adjustments. This stability reduces the ecological necessity for *S. oualaniensis* to undertake long-distance foraging migrations. Therefore, we infer that the population in this region primarily consists of short-distance moving groups driven by monsoon-related environmental forcing, with the distributional gravity center shifting latitudinally in accordance with seasonal oscillations of suitable habitats.

### 4.3. Autocorrelation Analysis

Conducting spatial autocorrelation analysis on the distribution of *S. oualaniensis* in the northwestern Indian Ocean allows us to determine whether the species exhibits a significant spatial structure across the study region [56,57]. From 2016 to 2024, Moran’s I values were consistently positive and statistically significant, indicating that *S. oualaniensis* displayed a pronounced clustered distribution rather than a random or dispersed pattern within the study area (Table 2) [58]. This aggregation tendency reflects the species’ collective response to specific environmental conditions, particularly its preference for oceanographic “hotspots” characterized by high productivity and intense physical dynamics [59,60]. During this period, the resource distribution exhibited positive skewness (Sk > 0) and high kurtosis (Ku > 3), suggesting that the region was dominated by extensive low-abundance areas, while high-abundance zones were sharply peaked and spatially concentrated. Such a distribution pattern further supports the conclusion that *S. oualaniensis* tends to aggregate in particular dynamic ocean environments—such as areas of elevated primary productivity, frontal zones, or enhanced upwelling—which provide richer food resources and thereby increase feeding efficiency, survival, and reproductive success. It is noteworthy that although the year 2016 exhibited extremely low abundance values (Ku = 0.52), the Moran’s I index remained relatively high. This indicates that *S. oualaniensis* formed extensive, contiguous low-abundance aggregation zones during that year, which may be associated with the ENSO event that occurred in 2016 [61]. In contrast, 2020 showed an exceptionally high kurtosis value (Ku = 110.04) but a relatively low Moran’s I index, suggesting that the resource distribution that year was highly patchy, with high-yield areas dominated by scattered extreme-value points. This pattern may be linked to the La Niña event that developed in 2020 [62]. The year 2020 followed immediately after the termination of the extreme positive Indian Ocean Dipole (IOD) event in 2019 and represented a transitional adjustment period before shifting into La Niña conditions in the latter half of the year. Such large-scale climate mode transitions are often accompanied by substantial restructuring of the upper-ocean environment, causing environmental fields (e.g., SST) to become highly fragmented [63].

Hotspot analysis revealed that from 2016 to 2023, the fishing-ground hotspots gradually shifted northeastward and exhibited a continuous expansion trend, but a marked contraction occurred in 2024. This turning point is likely associated with the superposition of extreme climate events. According to NOAA data, a strong El Niño event and a positive Indian Ocean Dipole (IOD) occurred simultaneously in this region from late 2023 to early 2024, leading to elevated sea surface temperatures and reduced precipitation, which consequently lowered the habitat suitability of the fishing grounds. Previous studies have shown that the suitable habitat area of *S. oualaniensis* in the northwestern Indian Ocean is largest during La Niña events and smallest during El Niño events, and that its habitat tends to shift northward under El Niño conditions. This is highly consistent with the pronounced reduction in hotspot areas and northward movement of the distributional centroid observed in 2024 in this study [64]. Both the IOD and El Niño can induce anomalous variations in Chl-a concentrations across different regions of the Indian Ocean. In particular, the IOD can suppress the increase in Chl-a near the thermocline, thereby altering overall oceanic productivity levels [65].

## 5. Conclusions

Based on fishery data and multi-source oceanographic remote-sensing datasets from 2016 to 2024, this study employed a GAM model, gravity-center trajectory analysis, and spatial autocorrelation methods to systematically reveal the spatial distribution patterns of *S. oualaniensis* in the northwestern Indian Ocean and their responses to multi-scale environmental variability. The results show that from 2016 to 2024, the catch of *S. oualaniensis* exhibited a continuous increasing trend, with the most rapid growth occurring in 2019. The species displayed pronounced spatiotemporal heterogeneity, with high-yield areas consistently concentrated within 61–65° E and 14–19° N. The fishing-ground gravity center shifted northeastward, forming a distinct “southwest–northeast” spatial pattern with clear directional aggregation. Spatial autocorrelation analysis further confirmed a significant clustered distribution, and the strong positive skewness and leptokurtic characteristics in high-abundance zones indicate the species’ strong dependence on regional oceanographic dynamical hotspots. Moreover, *S. oualaniensis* abundance exhibited evident hot-spot and cold-spot structures: from 2016 to 2023, hot spots expanded and migrated northeastward, whereas in 2024 they contracted sharply—likely influenced by the combined effects of the El Niño and positive IOD events.

Of course, in addition to environmental drivers, the intensification of human fishing activities is also an important factor contributing to the increase in squid yield. With the ongoing expansion of fisheries development in the Indian Ocean, the continuous growth of fishing effort, the widening of fishing grounds, and the improvement of fishing technologies have directly promoted the rise in total catch. Moreover, in the context of habitat contraction caused by extreme climate events, sustained high fishing pressure may further exacerbate resource instability. Therefore, it is recommended that future fisheries management fully consider the dual driving mechanisms of “climate–human activities” and appropriately regulate fishing intensity during environmentally unfavorable years to achieve the sustainable utilization of the resource. In particular, given the persistent northeastward shift in the centroid of fishing grounds, adaptive and spatially explicit management strategies should be implemented. For example, fishing areas should be dynamically adjusted in response to the northward expansion of high-abundance zones; enhanced monitoring and regulation of fishing effort should be applied to newly emerging northern fishing grounds; and during extreme climate events (such as El Niño episodes and positive phases of the Indian Ocean Dipole), long-distance unidirectional voyages should be reduced in favour of increased mesoscale searching and cooperative operations among vessels. Such targeted measures would help mitigate the risk of resource overexploitation under conditions of habitat contraction and enhance the overall resilience of the *Sthenoteuthis oualaniensis* resource in the northwestern Indian Ocean.

## Figures and Tables

**Table 1:** Variable VIF testing results.

Variable	Year	Latitude	Longitude	Chl-a	SST	SSH	SSS	UO	VO
VIF value	1.28	1.269	2.084	1.716	2.824	2.126	1.273	1.162	1.15

**Table 2:** Global spatial autocorrelation parameters for the annual catch of *S. oualaniensis*.

Year	Mean	SD	Skwenss	Kurtosis	CV	Moran’s I	Z-Score	*p*-Values
2016	1.25	1.21	1.01	0.52	0.97	0.58	5.04	0.00
2017	5.05	7.46	2.08	3.78	1.48	0.30	10.79	0.00
2018	5.94	7.61	3.05	10.55	1.28	0.13	6.75	0.00
2019	8.26	7.56	1.93	6.28	0.92	0.15	11.31	0.00
2020	5.01	5.37	5.42	110.04	1.07	0.16	13.85	0.00
2021	4.28	3.78	1.99	6.32	0.88	0.43	15.83	0.00
2022	5.22	4.27	1.96	5.29	0.82	0.61	23.18	0.00
2023	4.93	4.05	1.71	4.84	0.82	0.29	14.28	0.00
2024	3.63	4.07	2.83	13.79	1.13	0.70	48.07	0.00

**Table 3:** GAM selection based on AIC.

GAM	R^2^	AIC	Explanation Rate (%)
log(CPUE + 0.1)~s(Year)	0.237	4819.917	23.9
log(CPUE + 0.1)~s(Year) + s(Longitude)	0.466	4193.471	46.8
log(CPUE + 0.1)~s(Year) + s(Longitude) + s(Latitude)	0.480	4148.185	48.3
log(CPUE + 0.1)~s(Year) + s(Longitude) + s(Latitude) + s(Chl-a)	0.492	4109.596	49.7
log(CPUE + 0.1)~s(Year) + s(Longitude) + s(Latitude) + s(Chl-a) + s(SST)	0.509	4054.524	51.3
log(CPUE + 0.1)~s(Year) + s(Longitude) + s(Latitude) + s(Chl-a) + s(SST) + s(SSS)	0.512	4044.422	51.8
log(CPUE + 0.1)~s(Year) + s(Longitude) + s(Latitude) + s(Chl-a) + s(SST) + s(SSS) + s(SSH)	0.518	4025.721	52.3
log(CPUE + 0.1)~s(Year) + s(Longitude) + s(Latitude) + s(Chl-a) + s(SST) + s(SSS) + s(SSH) + s(UO)	0.530	3981.758	53.6
log(CPUE + 0.1)~s(Year) + s(Longitude) + s(Latitude) + s(Chl-a) + s(SST) + s(SSS) + s(SSH) + s(UO) + s(VO)	0.537	3962.039	54.4

**Table 4:** ANOVA of the optimal GAM.

Parameter	df	F	*p* Value
year	4.000	172.811	2 × 10^−16^
latitude	3.670	167.040	2 × 10^−16^
longitude	3.869	13.852	2 × 10^−16^
Chl-a	3.581	7.214	3.24 × 10^−5^
SST	3.787	27.207	2 × 10^−16^
SSS	1.000	34.839	2 × 10^−16^
SSH	3.920	5.436	2.41 × 10^−4^
UO	3.524	11.980	2 × 10^−16^
VO	3.232	7.212	6.34 × 10^−5^

## Data Availability

The original contributions presented in the study are included in the article, and further inquiries can be directed to the corresponding authors.
